# Molecular signatures define two main classes of meningiomas

**DOI:** 10.1186/1476-4598-6-64

**Published:** 2007-10-15

**Authors:** Lucia Helena Carvalho, Ivan Smirnov, Gilson S Baia, Zora Modrusan, Justin S Smith, Peter Jun, Joseph F Costello, Michael W McDermott, Scott R VandenBerg, Anita Lal

**Affiliations:** 1 Brain Tumor Research Center, Department of Neurological Surgery, University of California, San Francisco, CA-94143, USA; 2 Department of Molecular Biology, Genentech, Inc., South San Francisco, California, 94080, USA; 3 Physiology Department, Santa Casa de São Paulo, School of Medicine, São Paulo SP, Brazil

## Abstract

**Background:**

Meningiomas are common brain tumors that are classified into three World Health Organization grades (benign, atypical and malignant) and are molecularly ill-defined tumors. The purpose of this study was identify molecular signatures unique to the different grades of meningiomas and to unravel underlying molecular mechanisms driving meningioma tumorigenesis.

**Results:**

We have used a combination of gene expression microarrays and array comparative genomic hybridization (aCGH) to show that meningiomas of all three grades fall into two main molecular groups designated 'low-proliferative' and 'high-proliferative' meningiomas. While all benign meningiomas fall into the low-proliferative group and all malignant meningiomas fall into the high-proliferative group, atypical meningiomas distribute into either one of these groups. High-proliferative atypical meningiomas had an elevated median MIB-1 labeling index and a greater frequency of copy number aberrations (CNAs) compared to low-proliferative atypical meningiomas. Additionally, losses on chromosome 6q, 9p, 13 and 14 were found exclusively in the high-proliferative meningiomas. We have identified genes that distinguish benign low-proliferative meningiomas from malignant high-proliferative meningiomas and have found that gain of cell-proliferation markers and loss of components of the transforming growth factor-beta signaling pathway were the major molecular mechanisms that distinguish these two groups.

**Conclusion:**

Collectively, our data suggests that atypical meningiomas are not a molecularly distinct group but are similar to either benign or malignant meningiomas. It is anticipated that identified molecular and CNA markers will potentially be more accurate prognostic markers of meningiomas.

## Background

Meningiomas account for ~30% of all primary central nervous system tumors [1]. These tumors are classified into three WHO Grades based on histopathological criteria. Approximately 80% of these tumors are WHO Grade 1 (benign), while the remaining 20% are either WHO Grade 2 (atypical) or Grade 3 (malignant) tumors [2,3]. Treatment options for meningioma patients are limited to traditional forms of cancer therapy, i.e. surgical resection and radiation therapy. Meningioma patients face extremely variable clinical outcomes [4]. While benign meningioma patients can often be cured by surgical resection alone, atypical and malignant meningioma patients have worse clinical outcomes [4]. Some benign meningiomas recur despite complete resection and/or occur in locations that are not amenable to complete resection [5]. While local 5-year recurrence rates are 5% for benign meningiomas, they are about 40% for totally resected atypical meningiomas. Malignant meningiomas are associated with high local recurrence rates and patients have an overall median survival of less than 2 years [6]. The histopathological based grading system has proved useful in predicting prognosis and in defining treatment regimens for meningiomas [7]. However, there remains considerable variability in clinical outcomes within each grade, especially among atypical meningiomas. Existing criteria do not adequately predict the rates of tumor growth or the likelihood of tumor recurrence. Also, the histopathological classification system provides no information on underlying molecular alterations. In contrast, a molecular based classification system has the likelihood of being a better prognostic indicator and is useful for identifying alterations in pathways and networks that drive tumor progression and growth [8]. The information obtained can potentially be translated into more effective and less toxic targeted therapies [8].

Meningiomas are cytogenetically well characterized but are molecularly less defined [3]. Mutation of the neurofibromatosis 2 (NF2) gene on chromosome 22q12 is a frequent and early event in meningioma tumorigenesis [9]. Atypical and malignant meningiomas have more complex genetic alterations with losses of the G_1_-S phase cell cycle checkpoint regulators, CDKN2A and CDKN2B, and p14^ARF ^on chromosome 9p contributing to more aggressive meningioma phenotypes [10]. High-throughput techniques have been used to analyze the global genome and transcriptome profiles of meningiomas with interesting insights into the biology of meningiomas [11-15]. However, these studies have either surveyed limited number of genes [14,15], analyzed limited number of samples [11], focused on differences arising due to meningioma location [12] or only investigated chromosomal aberrations [13].

The purpose of this study was to identify unique molecular signatures that characterize the different grades of meningiomas and to identify underlying molecular mechanisms driving meningioma tumorigenesis. We have performed a comprehensive analysis of the expression pattern of over 47,000 transcripts in 23 primary meningiomas of the three histopathological grades using oligonucleotide microarrays. Copy number aberrations (CNA) in the same 23 tumors were defined using array comparative genomic hybridization (aCGH). We find that meningiomas of all three grades fall into two main molecular groups designated 'low-proliferative' and 'high-proliferative' meningiomas. Gain of cell proliferation markers and loss of transforming growth factor beta (TGF-β) signaling are the main molecular mechanisms that distinguish benign low-proliferative tumors from malignant high-proliferative tumors.

## Results

### Global gene expression patterns

Twenty-three meningioma samples of the three histopathological grades were profiled using gene expression microarrays to identify molecular signatures unique to each group and to unravel underlying molecular mechanisms of meningioma progression. We performed pair-wise comparisons between the three WHO grades of meningiomas using SAM analysis. 28 genes were differentially expressed between Grade 1 and 2 meningiomas and no genes were differentially expressed between Grade 2 and Grade 3 meningiomas using a criterion of q < 0.05 and fold > 2. In contrast, 1,212 genes (q < 0.05; fold > 2) were differentially expressed between Grade 1 and Grade 3 meningiomas. Since SAM analysis utilizes the median expression of individual genes within each group to calculate fold changes, this result suggests that Grade 2 meningiomas were the most heterogeneous group with expression profiles of individual tumors matching both Grade 1 and Grade 3 meningiomas.

**Figure 1:**
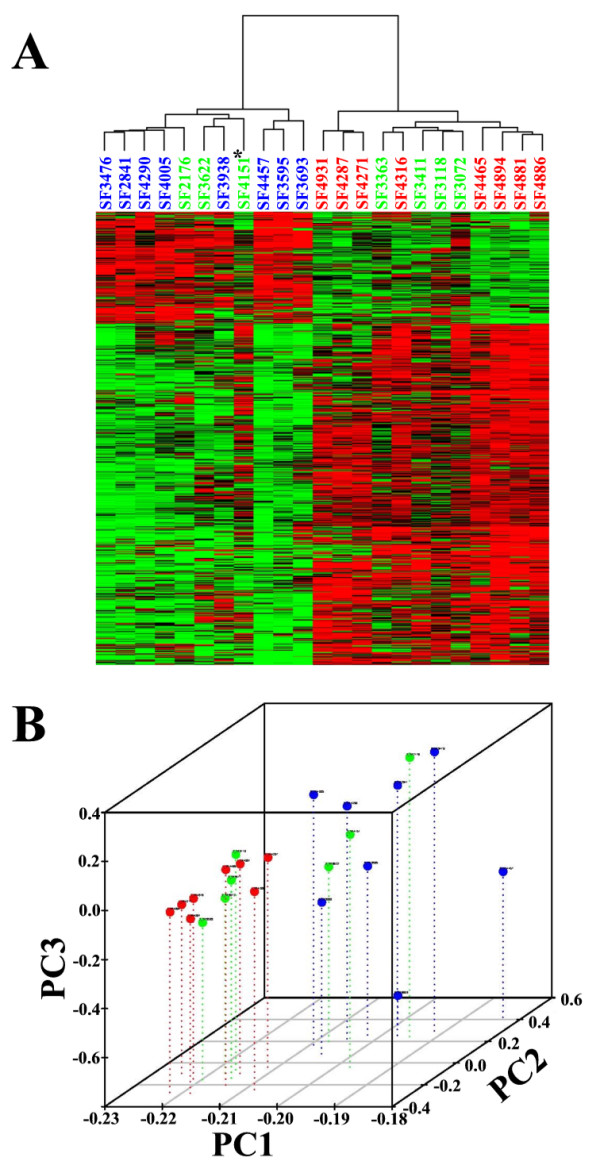
**Gene expression profiling of meningiomas**. A) Unsupervised hierarchical clustering of expression data derived from 23 primary meningioma tumors of the three histopathological grades was performed. Each column represents one case and each row represents the expression value of an individual probeset. Expression values are color coded as follows: higher (red), lower (green) or close (black) to the median expression value of each probeset. Tumor case numbers are listed above each column and are color-coded by histopathological grade. Red, Grade 1; Green, Grade 2; and Blue, Grade 3. The classification tree on top suggests two major molecular subgroups of meningiomas. The asterisk refers to the Grade 2 meningioma with a unique expression pattern. B) Principal component analysis of the meningioma expression profiles. Distribution of the 23 primary meningioma tumors along the three calculated principal components, PC1, PC2 and PC3. Tumor cases are color coded by histopathological grade. Red, Grade 1; Green, Grade 2; and Blue, Grade 3.

To identify meningioma tumors with similar expression patterns, we performed unsupervised clustering on the microarray data. This analysis yielded two main branches of a dendrogram (Figure 1A). The left branch was designated the 'low-proliferative' group because it contained all eight benign meningiomas while the right branch was designated the 'high-proliferative' group because it contained all eight malignant meningiomas. The atypical meningiomas distributed into either group, with four cases consistently falling into the 'low-proliferative' group and two cases consistently falling into the 'high-proliferative' group. One atypical case, SF4151, clustered with either group depending on the filtering criteria used and was considered unique (Figure 1A). Similar results were obtained when a principal component analysis of the expression data was performed (Figure 1B). Once again, all eight benign meningiomas and four atypical meningiomas clustered into one group while all eight malignant meningiomas and two atypical meningiomas clustered into a second group. The atypical meningioma, SF4151, was an outlier, not truly fitting into either group. Collectively, this data suggests that meningiomas of all three histopathological groups fell into two main molecular subtypes.

### Histopathological characteristics

Since the Grade 2 meningiomas in our dataset were molecularly similar to either Grade 1 or Grade 3 meningiomas, we assessed their histopathological characteristics in greater detail (Table 1). Meningiomas are classified as Grade 2 meningiomas if they have increased mitotic activity and/or have the presence of three of the following five criteria: increased cellularity, high nuclear to cytoplasmic ratio, prominent nucleoli, cellular pattern loss and foci of spontaneous necrosis [16,17]. The presence of brain invasion is also used as a criterion for a Grade 2 classification [16,17]. All Grade 2 meningiomas in our dataset fit these histopathological criteria (Table 1). Also, there were no specific histopathological criteria that could distinguish the 'low proliferative' Grade 2 meningiomas from the 'high proliferative' Grade 2 meningiomas.

**Table 1:** Histopathological Characteristics of Meningioma Tumors

	**SF4271**	**SF4287**	**SF4316**	**SF4465**	**SF4881**	**SF4886**	**SF4894**	**SF4931**	**SF3072**	**SF3118**	**SF3363**	**SF3411**	**SF4151**	**SF2176**	**SF3622**	**SF2841**	**SF3476**	**SF3595**	**SF3693**	**SF3938**	**SF4005**	**SF4290**	**SF4457**
Grade	**BENIGN (GRADE 1)**								**ATYPICAL (GRADE 2)**							**MALIGNANT (GRADE 3)**							
		
Molecular Subgroup	**LOW-PROLIFERATIVE**												*	**HIGH-PROLIFERATIVE**									
	
Embolized	No	Y	Y	No	No	No	No	Y	No	Y	No	Y	No	No	No	Y	Y	No	N	Y	Y	Y	No
	
Histological Subtype	M	T	M	M	Fi	T	T	T						CC									
Mitotic Index	N	N	N	N	N	E/F	E/F	N	N	N	N	N	E/F	N	E/F	E	E/F	E	E	E	E	E	E
Increased Cellularity	E/F	N	N	N	N	N	E/F	N	N	E/F	E/F	E	E/F	E	E	E	E	E	E	E	E	E	E
Nuclear/Cytoplasmic Ratio	E/F	N	E/F	N	N	E/F	E/F	N	E	E	E	E	N	E/F	E/F	E	F	E	E	E	E	E	E
Prominent Nucleoli	E/F	N	E/F	N	N	E/F	E/F	N	E	E	E	E	N	E/F	E/F	E	F	E	E	E	E	E	E
Cellular Pattern Loss	E/F	N	N	N	N	N	E/F	N	N	E/F	E/F	E	E/F	E	E	E	E/F	E	E	E	E	E	E
Necrosis	E/F	E/F	E/F	N	N	N	N	N	N	N	N	E	N	N	E	E	E	E	E/F	E	E	E	E
Brain Invasion									Y							Y					Y		
MIB-1	1.5	1.6	2.0	0.9	0.8	0.1	1.3	1.9	5.9	4.2	3.7	7.0	7.0	7.7	8.5	10.9	11.3	17.5	20.3	16.9	7.5	7.5	23.3

No = No; Y = Yes; M = Meningothelial; T = Transitional; Fi = Fibroblastic; CC = Clear Cell Characteristics N = Not Elevated or Absent; E = Elevated; F = Focal; ^§^E indicates a mitotic index of >4 mitosis/10 high powered field while N indicates <4 mitosis/10 high powered field; * Refers to the Grade 2 meningioma that was an outlier

Increased MIB-1 labeling indices have been associated with an increased risk of recurrence in meningiomas and are sometimes used as an accessory to grading meningiomas [18]. We therefore assessed the MIB-1 labeling indices of the meningioma dataset (Table 1). As anticipated, the median MIB-1 labeling indices increased with histopathological grade, with values of 1.4 for Grade 1, 7.0 for Grade 2 and 14.1 for Grade 3 meningiomas. While the MIB-1 labeling indices of the high proliferative Grade 2 meningiomas (median = 8.1; Range = 7.5–8.0) were higher than the low-proliferative Grade 2 meningiomas (median = 5.1; Range = 3.7–7.0), larger sample numbers are required to confirm this trend.

### Clinical outcome

Clinical patient data of the 23 profiled meningiomas are summarized in Table 2. Although the sample numbers are small, we assessed whether the low-proliferative atypical meningiomas had a better clinical outcome when compared to the high-proliferative atypical meningiomas. Clinical outcome was measured as the disease status at 2 years post-surgery. Once again, SF4151, the atypical meningioma with a unique profile was not included in the analysis. Interestingly, this patient was dead at 2 years post-surgery. As anticipated, benign meningioma patients had a better clinical outcome when compared to malignant meningiomas. Only 1 in 8 benign meningiomas had recurred, while 7 in 8 resected malignant meningiomas had recurred and 5 of these patients were dead at two years. One patient belonging to the low-proliferative atypical meningiomas was lost to follow-up. Among the remaining 5 atypical meningiomas, the only patient that had recurrent disease was a high-proliferative atypical meningioma. This is consistent with the high-proliferative atypical meningiomas having a more aggressive clinical course when compared to the low-proliferative atypical meningiomas. However, larger sample numbers are needed to confirm this trend.

**Table 2:** Patient Clinical Data of the 23 Profiled Meningiomas

	**SF4271**	**SF4287**	**SF4316**	**SF4465**	**SF4881**	**SF4886**	**SF4894**	**SF4931**	**SF3072**	**SF3118**	**SF3363**	**SF3411**	**SF4151**	**SF2176**	**SF3622**	**SF2841**	**SF3476**	**SF3595**	**SF3693**	**SF3938**	**SF4005**	**SF4290**	**SF4457**
**Grade**	**BENIGN (GRADE 1)**								**ATYPICAL (GRADE 2)**							**MALIGNANT (GRADE 3)**							
		
**Molecular Subgroup**	**LOW-PROLIFERATIVE**												*	**HIGH-PROLIFERATIVE**									
	
**Embolized**	**No**	**Y**	**Y**	**No**	**No**	**No**	**No**	**Y**	**No**	**Y**	**No**	**Y**	**No**	**No**	**No**	**Y**	**Y**	**No**	**N**	**Y**	**Y**	**Y**	**No**
	
Sex	F	F	F	F	F	F	F	F	F	F	F	F	F	F	M	F	F	F	F	F	M	F	F
Age	58	57	74	72	56	51	65	35	39	62	63	47	64	60	43	83	71	52	52	58	74	58	67
Location	C	C	C	S	C	C	S	S	C	C	I	s	S	C	F	T	T	C	C	C	C	C	C
Primary or Recurrent	P	P	P	P	P	P	P	P	P	P	P	P	P	P	P	P	P	R	R	P	P	P	R
Extent of Resection	G	G	Su	G	G	G	G	G	G	Su	G	G	G	G	G	Su	Su	Su	Su	G	G	G	G
Disease Status@2yr	NR	NR	R	NR	NR	NR	NR	NR	NR	NR	U	NR	D	NR	R	D	R	D	D	R	D	NR	D

F = Female; M = Male; C = Convexity; S = Skull Base; I = Intraventricular; s = spinal; F = Falx; T = Tentorium; P = Primary; R = Recurrent; G = Gross Total Resection; Su = Sub Total Resection; NR = Non-Recurrent; R = Recurrent; D = Dead; U = Unknown;* Refers to the Grade 2 meningioma that was an outlier

### aCGH changes

**Figure 2:**
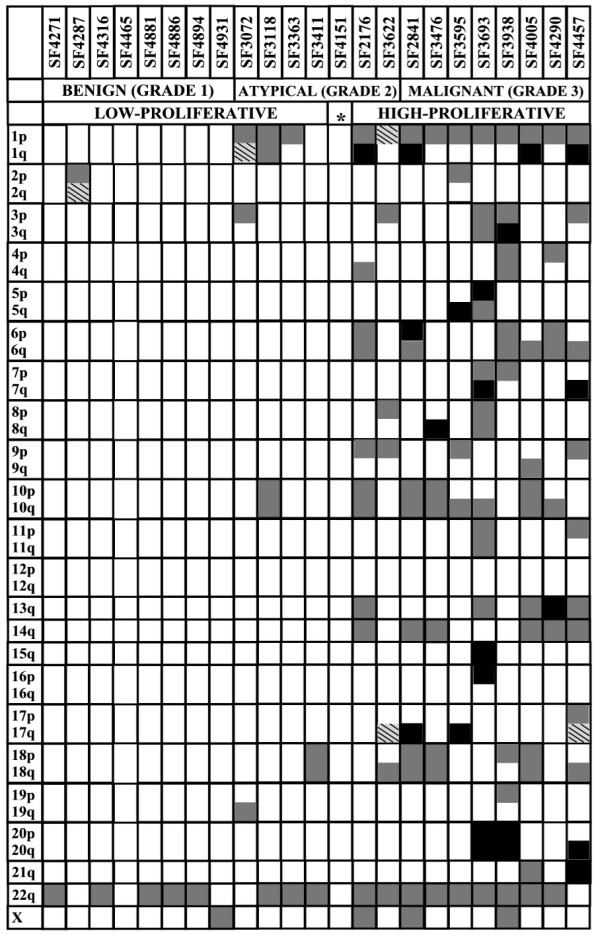
**Copy number aberrations of meningiomas**. Array-comparative genomic hybridization data derived from 23 primary meningioma tumors were processed to identify loss (grey boxes), gain (black boxes) or both loss and gain (patterned boxes) of part or all of a given chromosomal arm. Each column represents one case and each row represents the indicated chromosomal arm. The WHO Grades and the molecular subgroups of the individual cases are indicated below the case numbers. The asterisk refers to the Grade 2 meningioma with a unique expression pattern.

To correlate expression data with genome copy number alterations, we performed aCGH on the identical set of 23 meningiomas used for the gene expression analysis. Chromosomal alterations were observed in 7 of 8 (88%) benign, 6 of 7 (86%) atypical and 8 of 8 (100%) malignant meningiomas (Figure 2). Interestingly, while chromosomal gains were observed, no gene amplifications (>4 copy numbers) were detected in this set of 23 meningiomas. Globally, chromosomal losses were far more frequent than chromosomal gains. As anticipated, the number of total chromosomal alterations in individual tumors increased with the histopathological grade (Figure 2). The average number of chromosomal alteration was 1 for benign, 5 for atypical and 11.6 for malignant meningiomas. The most frequent deletion was loss of chromosome 22 (78.3%), followed by losses of regions of chromosome 14q (60.9%) and chromosome 1p (56.5%). Other chromosomal changes that were found in greater than 20% of the tumors were losses on chromosome 3p, 6q, 10, 14q and 18 and gains on chromosome 1q.

We next analyzed if the two main molecular groups of meningiomas had specific chromosomal alterations. SF4151, the atypical meningioma that had a unique expression profile, was not included in this analysis. SF4151 had no detectable gross chromosomal aberrations, an unusual characteristic for an atypical meningioma (Figure 2). The two atypical meningiomas that were similar to malignant meningiomas did have a greater number of total chromosomal alterations when compared to the atypical meningiomas that were similar to the benign meningiomas. Several deletions/gains were found exclusively in the high-proliferative meningioma subgroup. Losses on chromosome 6q, 9p, 13 and 14 were all found at a frequency of greater than 40% in the high-proliferative subgroup.

### Correlation between CNAs and gene expression

**Figure 3:**
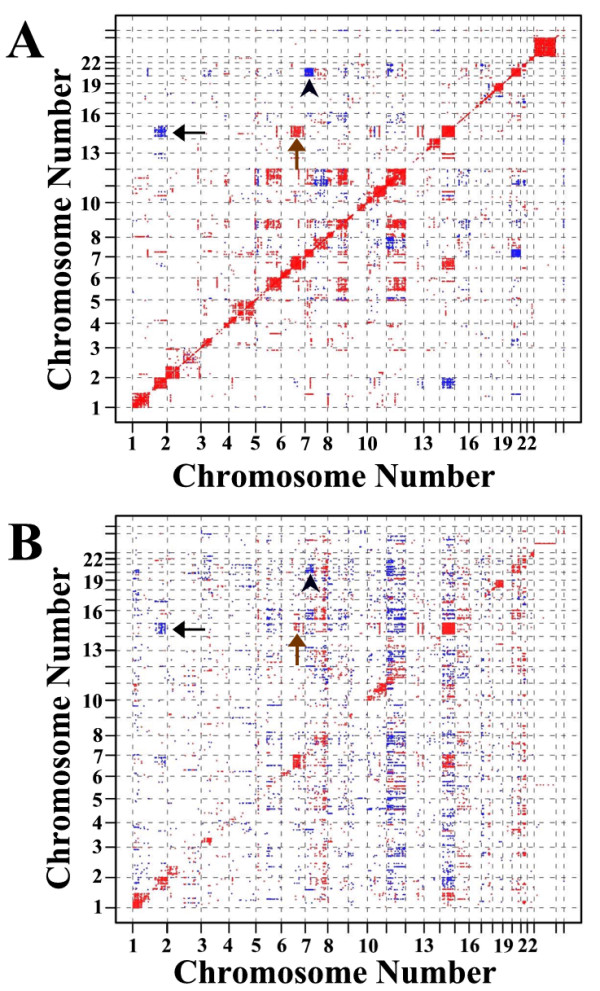
**Correlation matrices of genome copy number and gene expression in meningiomas**. A, Pearson's correlations among DNA copy numbers (Panel A) and between DNA copy number and gene expression (Panel B) are plotted as a function of chromosomal location. Only correlations greater than 0.82 (red) and less than -0.82 (blue) are shown on the intensity map. The association between loss on chromosome 14 and gain on chromosome 1q (black arrow), loss on chromosome 14 and loss on chromosome 6q (brown arrow) and loss on chromosome 7p and gain on chromosome 20 (black arrowhead) are indicated.

We evaluated correlations among CNAs by chromosomal location, and also between CNAs and expression levels of genes mapping to the same locus. In addition to the anticipated correlation between CNAs in neighboring regions on the same chromosomes (Figure 3A; diagonal line), several additional correlations between CNAs on different chromosomes were observed (Figure 3A). While CNAs and gene expression levels at the same locus were positively correlated, this relationship varied along the genome (Figure 3B; diagonal line). Once again, positive and negative DNA-RNA correlations away from the diagonal of the matrix plot were observed. Positive correlation between chromosome 14 and 6q, and the inverse correlations between chromosome 14 and 1q and chromosomes 20 and 7q were maintained among CNAs and between CNAs and gene expression (Figure 3). This suggests that the mechanism of gene expression changes was due to DNA copy number alterations.

### Molecular mechanisms underlying meningioma progression

A subset of the genes differentially expressed between Grade 1 and 3 meningiomas (q < .05; fold > 4) are listed [see Additional File 1 and Additional File 2]. This included novel genes that have not been implicated in meningioma pathogenesis and several genes that have previously been reported as altered in meningiomas. It is well established that loss of the progesterone receptor (PR) is observed in Grade 3 meningiomas compared to Grade 1 meningiomas [19,20]. In our dataset, transcript levels of *PR *were reduced in Grade 3 meningiomas. In addition, we found induction of *IGFBP3*, *CENPF*, *CKS2 *and reduction of *LTBP2*, *PTPRF *in high-grade meningiomas as previously reported [15].

To identify molecular mechanisms that underlie Grade 1 to Grade 3 meningioma progression, we analyzed differentially expressed genes (p < 0.05 and fold > 2) using the GenMAPP software package [21]. This program analyzes gene expression in the context of known biological pathways and functions by mapping genes to Gene Ontology and Contributed MAPPs and calculating a confidence level based on the percentage of altered genes assigned to each pathway [22,23]. Biological pathways, cellular components and functions that were significantly altered between Grade 1 and Grade 3 meningiomas included cell cycle and mitosis related genes, extracellular matrix components and the TGF-β signaling pathway (Table 3).

**Table 3:** Pathways significantly altered between grade 1 and grade 3 meningiomas by GenMAPP analysis of genes altered^a^

Database	Mapp or GO name	P value	Number Changed^b^	Percent Changed^c^
Local Mapp	Hs 2 Tissues Endocrine and CNS	0.000	25	24.3
	M phase of mitotic cell cycle	0.001	26	21.7
	extracellular matrix (sensu Metazoa)	0.008	32	18.6
	Hs TGF Beta Signaling Pathway	0.008	14	26.9
	M phase	0.010	29	18.8
	Hs Focal adhesion KEGG	0.012	33	17.6
	Hs 1 Tissue Embryonic Stem Cell	0.044	12	25.5
				
Gene Ontology	spindle organization and biogenesis	0.000	11	57.9
	extracellular matrix	0.000	50	19.6
	extracellular matrix (sensu Metazoa)	0.001	49	19.7
	cell division	0.017	33	22.1
	extracellular region	0.023	117	12.1
	spindle	0.024	11	39.3
	cell adhesion	0.025	73	13.7
	mitotic cell cycle	0.026	33	19.2
	mitosis	0.049	26	21.1

^a^The results were obtained using a criterion of p ≤ 0.05 and fold-difference ≥ 2. ^b^The number of genes mapping to a particular pathway that are altered is listed. ^c^The percentage of total genes belonging to a particular pathway that are altered is listed.

### TGF-β pathway alterations in meningiomas

**Figure 4:**
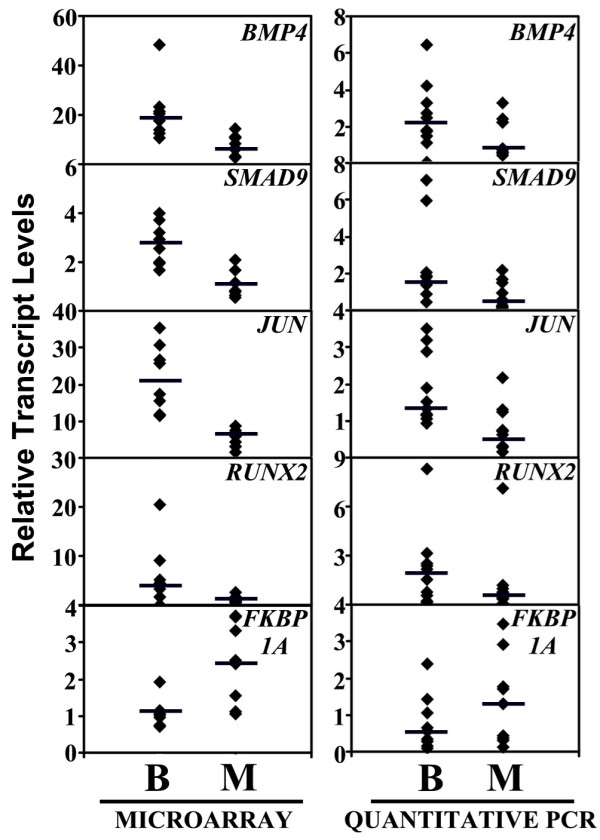
**Transcript abundance of components of the transforming growth factor-β signaling pathway in meningiomas**. Relative transcript numbers of *BMP4*, *SMAD9*, *JUN*, *RUNX2 *and *FKBP1A *in individual Grade 1 (B) and Grade 3 (M) meningioma tumors (filled diamonds) are plotted. The data was derived either from microarray or from quantitative PCR analysis. The median expression level of the genes in each group is marked by a horizontal line.

Fourteen genes that participate in TGF-β signaling were differentially expressed between Grade 1 and Grade 3 meningiomas (Table 4). The majority of these components had reduced expression levels in Grade 3 meningiomas, suggesting loss of TGF-β signaling as a mechanism contributing to the development of higher-grade meningiomas. In the case of five genes, we confirmed differences at the transcript levels using quantitative PCR in 10 Grade 1 (5 cases represented in the microarray and 5 independent cases) and 10 Grade 3 (5 cases represented in the microarray and 5 independent cases) meningiomas (Figure 4). In line with the microarray data, the PCR data showed a decrease in the median expression level of *BMP4, SMAD9, JUN, RUNX2 *and an increase in the median expression level of *FKBP1A *in Grade 3 meningiomas when compared to Grade 1 meningiomas.

**Table 4:** TGF-β Signaling Pathway Components Significantly Altered

Gene Symbol	Gene Name	Probe Set ID^a^	Fold Change^b^
*RUNX2*	runt-related transcription factor 2	232231_at	-6.1
*THBS1*	thrombospondin 1	201108_s_at	-5.3
*JUN*	jun oncogene	201465_s_at	-4.0
*NOG*	noggin	231798_at	-3.6
*FST*	follistatin	226847_at	-3.6
*SMAD7*	SMAD family member 7	204790_at	-3.0
*BMP4*	bone morphogenetic protein 4	211518_s_at	-2.6
*SMAD9*	SMAD family member 9	206320_s_at	-2.5
*SKI*	v-ski sarcoma viral oncogene homolog	204270_at	-2.4
*TGFBR3*	transforming growth factor, beta receptor III	204731_at	-2.3
*FOS*	v-fos FBJ murine osteosarcoma viral oncogene homolog	201809_s_at	-2.0
*ENG*	endoglin	201809_s_at	-2.0
*FKBP1A*	FK506 binding protein 1A	210187_at	2.2
*SPP1*	secreted phosphoprotein 1	1568574_x_at	2.5

^a^The corresponding affymetrix probe set ID that was altered is listed. ^b^A negative fold change indicates a decrease in grade 3 meningiomas compared to grade 1 meningiomas and a positive number indicates an increase in grade 3 meningiomas compared to grade 1 meningiomas.

## Discussion

We have profiled gene expression changes and genomic CNAs of primary meningioma tumors belonging to the three WHO malignancy grades. Independent methods of analysis of the expression data revealed that meningiomas of all three grades can be classified into two main molecular groups. We have designated these molecular groups as the low-proliferative and the high-proliferative meningiomas to reflect the fact that all the slower-growing benign tumors fell into the former group and all the faster-growing malignant tumors fell into the latter group. Atypical meningiomas did not have a unique molecular signature of their own. Instead, their molecular profiles matched those of either benign or malignant meningiomas. Clinically, atypical meningiomas demonstrate a wide variability in clinical behaviour, with some tumors exhibiting growth patterns similar to benign meningiomas and others having poor clinical outcomes paralleling those of malignant meningiomas [4]. Our data suggests that molecular signatures could distinguish the slower-growing atypical meningiomas from the more aggressive ones.

Previous attempts at profiling the expression pattern of meningiomas were unable to reliably distinguish the different grades of meningiomas and identify specific expression patterns that were representative of each grade [14,15]. In these prior studies, the two molecular classes of meningiomas were probably not clearly distinguishable because of the considerably fewer number of genes surveyed. The considerable morphologic and biological heterogeneity of meningiomas was thought to be responsible for the lack of consistent molecular profiles, and atypical and malignant meningiomas were grouped together and compared to benign meningiomas [14,15]. Interestingly, one of these studies did report that a set of genes could be used for a clear distinction between benign and malignant meningiomas [15].

Using histopathological criteria, atypical meningiomas were a distinct group and no specific criteria, except for MIB-1 labeling indices, could distinguish the low-proliferative atypical meningiomas from the high-proliferative atypical cases. Additional studies with larger numbers of samples are needed to verify the molecular classification scheme, confirm differences in the MIB-1 labeling indices and perform clinical correlates.

Cytogenetically, the profile of our meningioma dataset is similar to prior reports on meningiomas [13]. We have identified several cytogenetic changes including losses on chromosome 9p, 6q, 13 and 14 that were exclusively found in the high-proliferative meningiomas. Losses of chromosome 9p have previously been implicated in the malignant progression of meningiomas and has been associated with a poor prognosis in malignant meningiomas [24]. It is likely that one or more of these chromosomal regions contain tumor suppressors that are responsible for progression of low-proliferative meningiomas to high-proliferative meningiomas. Losses of cyclin dependent kinase inhibitors, CDKN2A and CDKN2B, have been implicated as the tumor suppressor genes on chromosome 9p responsible for malignant meningioma phenotypes [10,25].

We have focused on alterations between benign and malignant meningiomas since these histopathological groups are the ones that can be reliably distinguished. These altered genes are also potential candidates for markers that can classify atypical meningiomas into either the low-proliferative or the high-proliferative groups. Among these genes were several cell cycle related genes and components of the TGF-β signaling pathway. The role of the TGF-β signaling pathway in the pathogenesis and malignant progression of meningiomas is unclear. Normal meninges synthesizes and secretes all three isoforms of TGF-β and these are present in the cerebrospinal fluid at concentrations that activate TGF-β receptors [26,27]. TGF-β1 inhibits proliferation of meningeal and benign meningioma cells and this appears to be mediated by signaling through the SMAD 2/3 pathway [28]. Thus, it seems likely that TGF-β exerts an inhibitory effect on benign meningiomas and that loss of TGF-β signaling and/or resistance to the growth inhibitory effects of TGF-β results in progression to malignancy. Our expression data supports this hypothesis.

In summary, even though our sample numbers are limited, the combination of the large numbers of genes surveyed and several different types of analysis allowed the identification of two meningioma molecular classes. MIB-1 labeling data, cytogenetic data and the clinical data are all consistent with the classification into low-proliferative and high-proliferative meningiomas.

## Conclusion

We provide evidence for the existence of a two group molecular classification scheme for meningiomas based on molecular signatures. Our data suggests that molecular and CNA markers will be able to distinguish low-proliferative atypical meningiomas from high-proliferative atypical meningiomas and will potentially be more accurate predictors of atypical meningioma tumor growth than histopathological criteria. It is envisaged that, in the future, these markers will be used in conjunction with histopathological grading to determine the prognosis and treatment regimens of atypical meningioma patients.

## Methods

### Tumor samples

All human meningioma tumor samples were collected by the Neurological Surgery Tissue Bank using protocols approved by the UCSF Committee on Human Research. A neuropathologist (S.V.) graded each case using the revised 2000 WHO grading system [2]. A total of 23 (8 Grade I, 7 Grade II and 8 Grade III) meningiomas were used in the current analysis. H&E stained sections adjacent to the frozen meningioma tumor pieces were examined to confirm tumor histologies.

### RNA expression arrays

Total RNA was isolated from snap-frozen tumor samples using the RNAeasy Kit (Qiagen, Valencia, CA) following manufacturer's instructions and the quality of the RNA was confirmed using the RNA 6000 Nano kit (Agilent Technologies, Foster City, CA). Gene expression profiles for individual tumors were generated by hybridization to the Human Genome U133 Plus 2.0 oligonucleotide array (Affymetrix, Santa Clara, CA) using previously described protocols [29]. This array contains 54,000 probe sets and over 47,000 transcripts, including 38,500 well-characterized genes.

Expression measures were generated from raw probe level data (*CEL *files) using the robust multi-array average (RMA) function of the affy package [30,31]. The preprocessing involved background-adjustment, normalization and log transformation. Presence-absence calls for expression of probesets were made using the panp package [30]. Unsupervised hierarchical clustering was performed using Ward's linkage method with Euclidean distances and the hclust function in the R package. The data were filtered to limit the probe sets to probesets that had a standard deviation of greater than 0.5, which gave us 12,374 probe sets out of the total 54,675 represented on the array. Principal component analysis was performed using the princomp function of the R stats package.

Differentially expressed genes between histopathological grades were identified using the siggenes package [30]. Significance Analysis of Microarrays (SAM) two-class unpaired analysis was used to calculate p-values, q-values and fold changes in expression levels [32]. The calculated gene expression changes were analyzed in the context of known biological pathways and functions using GenMAPP 2.1 [21,22]. Significant associations with GO biological process, molecular function, cellular component groups and with contributed GenMAPP biological pathways were obtained with MAPPFinder 2.0 using the Hs-Std_20060526 database [23].

### aCGH

Genomic DNA was isolated from meningioma primary tumors and from normal whole blood from anonymous donors using the DNAeasy kit (Qiagen, Valencia, CA) following manufacturer's directions. The DNA was labeled and hybridized to arrays spotted with 2,464 mapped bacterial artificial chromosomes (BAC) covering the whole genome as described earlier [33]. The array images were processed using the SPOT custom software [34]. Briefly, relative ratios of tumor to normal DNA copy number for individual BACs were normalized by setting the value of the median relative ratio for that hybridization to 1. The data was then log transformed. Losses or gains for that tumor were scored as described earlier [35]. Copy number frequency maps were constructed by applying circular binary segmentation (CBS) algorithm to each aCGH profile and aggregating the CBS smoothed profiles from all the samples [36].

### aCGH and expression correlations

Individual BACs were mapped to the University of California, Santa Cruz genome database March 2006 freeze [37]. Probesets were mapped to chromosomal locations and the correlation between CNAs and gene expression was determined by calculating Pearson's correlations as previously described [38].

### Quantitative PCR

Quantitative PCR was performed on cDNA templates with the I-cycler machine (Bio-Rad, Hercules, CA) and SYBR Green I (Molecular Probes, Eugene, OR) using PCR conditions and data analysis as described earlier [11]. The primers used were as follows: *BMP4 *– 5'-TGGCTGTCAAGAATCATGGA and 5'-CTTCCCCGTCTCAGGTATCA; *FKBP1A *– 5'-CCTTTGCTCCTCCCATGTTA and 5'-CACATGCCAATTCCTTTCCT; *SMAD9 *– 5'-ACAGCAGCATCTTTGTGCAG and 5'-AAAGCCGTGGTGAACTGACT; *JUN *– 5'-GCAGCCCAAACTAACCTCAC and 5'-CAGGGTCATGCTCTGTTTCA and *RUNX2 *– 5'-CAGACCAGCAGCACTCCATA and 5'-CAGCGTCAACACCATCATTC. Primers specific for the housekeeping gene, beta-actin (5'-ACTCTTCCAGCCTTCCTTCC and 5'-CAGGAGGAGCAATGATCTTG), were used to verify the integrity of the cDNA and to normalize cDNA yields.

## Competing interests

The author(s) declare that they have no competing interests.

## Authors' contributions

LHC participated in the sample collections and RNA and DNA isolations and quality controls, carried out the quantitative PCR studies, participated in the design of the study and in the bioinformatics and helped to draft the manuscript. IS participated in the design of the study and carried out the bioinformatics. GSB participated in the sample collections and RNA and DNA isolations and quality controls and participated in the design of the study. ZM carried out the microarray analysis. PJ and JFC carried out the array CGH analysis. JSS and MWM participated in the design of the type of meningioma clinical data to be collected and collected the clinical data. SRV carried out the meningioma grading and the detailed histopathological characterizations. AL conceived of the study, and participated in its design and coordination and drafted the manuscript. All authors read, contributed to edits and approved the final manuscript.

## Supplementary Material

Additional file 1 Genes overexpressed in grade 3 compared to grade 1 meningiomas. The data includes a list of genes with more than 4-fold induction and q < 0.05 in expression levels in grade 3 compared to grade 1 meningiomas. Click here for file

Additional file 2 Genes reduced in grade 3 compared to grade 1 meningiomas. The data includes a list of genes with more than 4-fold reduction and q < 0.05 in expression levels in grade 3 compared to grade 1 meningiomas. Click here for file
