# Supplementary material for: Molecular signatures define two main classes of meningiomas
Source: Mol Cancer. 2007 Oct 15;6:64. doi: 10.1186/1476-4598-6-64 (PMC2173907; doi:10.1186/1476-4598-6-64)
Supplement: Additional file 1 — Genes overexpressed in grade 3 compared to grade 1 meningiomas. The data includes a list of genes with more than 4-fold induction and q < 0.05 in expression levels in grade 3 compared to grade 1 meningiomas. [file 1476-4598-6-64-S1.doc]

| **Additional Table 1 - Genes overexpressed in grade 3 compared to grade 1 meningiomas** | | | | |
| --- | --- | --- | --- | --- |
| Gene Symbol^a^ | Gene Name | Accession Number | Chr Loc^b^ | FC^c^ |
| *MGST1* | microsomal glutathione S-transferase 1 | NM_020300 | 12p12.3-p12.1 | 32.9 |
| *NMU* | neuromedin U | NM_006681 | 4q12 | 25.5 |
| *ALDH1A2* | aldehyde dehydrogenase 1 family, member A2 | AB015228 | 15q22.1 | 23.7 |
| *KRT14* | keratin, type 1, cytoskeletal 14 | BC002690 | 17q21.2 | 23.6 |
| *KLRC3* | killer cell lectin-like receptor subfamily C, member 3 | NM_002261 | 12p13.2 | 19.8 |
| *COL4A4* | collagen, type IV, alpha 4 | BC065843 | 2q36.3 | 15.9 |
| *AGR2* | anterior gradient 2 homolog (Xenopus laevis) | AF088867 | 7p21.1 | 15.5 |
| *EPHA3* | EPH receptor A3 | AF213459 | 3p11.2-p11.1 | 12.6 |
| *COL4A3* | collagen, type IV, alpha 3 (Goodpasture antigen) | NM_000091 | 2q36.3 | 12.5 |
| *ADAMTS5* | ADAM metallopeptidase with thrombospondin, type 1 motif, 5 | NM_007038 | 21q21.3 | 11.7 |
| *KISS1R* | G protein-coupled receptor 54 | Al819198 | 19p13.3 | 11.7 |
| *NLF1* | Nuclear localized factor 1 | BX648578 | 15q22.2 | 10.9 |
| *SOX11* | SRY (sex determining region Y)-box 11 | Al360875 | 2p25.2 | 10.7 |
| *ASPM* | asp (abnormal spindle)-like, microcephaly associated (Drosophila) | NM_018123 | 1q31.3 | 10.4 |
|  | hypothetical protein LOC440156 | BC013389 | 14q11.1 | 10.3 |
| *EPHA7* | EPH receptor A7 | BC027940 | 6q16.1 | 9.9 |
| *RRM2* | ribonucleotide reductase M2 polypeptide | BE966236 | 2p25.1 | 9.9 |
| *TOP2A* | topoisomerase (DNA) II alpha 170kDa | AL561834 | 17q21.2 | 9.9 |
| *CXCL1* | chemokine (C-X-C motif) ligand 1 (melanoma growth stimulating activity, alpha) | NM_001511 | 4q13.3 | 8.9 |
| *PDE4D* | Phosphodiesterase 4D, cAMP-specific (phosphodiesterase E3 dunce homolog, Drosophila) | BC035063 | 5q11.2 | 8.6 |
| *KLHL14* | kelch-like 14 (Drosophila) | AB037805 | 18q12.1 | 8.4 |
|  | cDNA clone IMAGE:4498449 | BG285837 | 21q22.2 | 8.0 |
| *KIAA0101* | KIAA0101 protein | NM_014736 | 15q22.31 | 7.6 |
| *C15orf48* | normal mucosa of esophagus specific 1 | AF228422 | 15q21.1 | 7.6 |
| *SYTL5* | synaptotagmin-like 5 | AW263497 | Xp11.4 | 7.5 |
| *NUSAP1* | nucleolar and spindle associated protein 1 | NM_016359 | 15q15.1 | 7.5 |
| *LMO3* | LIM domain only 3 (rhombotin-like 2) | AL050152 | 12p12.3-p12.1 | 6.9 |
| *CENPK* | centromere protein K | BC005400 | 5q12.3 | 6.7 |
| *IGFBP3* | insulin-like growth factor binding protein 3 | BF340228 | 7p13 | 6.7 |
| *SLITRK6* | SLIT and NTRK-like family, member 6 | AL137517 | 13q31.1 | 6.5 |
| *NCAPG* | chromosome-associated protein G | NM_022346 | 4p15.32 | 6.4 |
| *HMGA2* | high mobility group AT-hook 2 | NM_003483 | 12q14.3 | 6.3 |
| *CKS2* | CDC28 protein kinase regulatory subunit 2 | NM_001827 | 9q22.2 | 6.2 |
| *DTL* | denticleless homolog (Drosophila) | NM_016448 | 1q32.3 | 6.2 |
| *FAM70A* | family with sequence similarity 70, member A | NM_017938 | Xq24 | 6.2 |
| *PBK* | PDZ binding kinase | NM_018492 | 8p21.1 | 6.2 |
| *CDCA7* | cell division cycle associated 7 | AY029179 | 2q31.1 | 6.1 |
| *KNTC2* | kinetochore associated 2 | NM_006101 | 18p11.32 | 6.1 |
| *BUB1B* | BUB1 budding uninhibited by benzimidazoles 1 homolog beta (yeast) | NM_001211 | 15q15.1 | 6.0 |
| *KIF20A* | kinesin family member 20A | NM_005733 | 5q31.2 | 6.0 |
| *CDCA1* | cell division cycle associated 1 | AF326731 | 1q23.3 | 5.9 |
| *CEP55* | Centrosome protein 55 | NM_018131 | 10q23.33 | 5.8 |
| *PI3* | peptidase inhibitor 3, skin-derived | NM_002638 | 20q13.12 | 5.8 |
| *TPX2* | TPX2, microtubule-associated, homolog (Xenopus laevis) | AF098158 | 20q11.21 | 5.8 |
| *NEFL* | Neurofilament, light polypeptide 68kDa | BF055311 | 8p21.2 | 5.7 |
| *DLG7* | discs, large homolog 7 (Drosophila) | NM_014750 | 14q22.3 | 5.6 |
| *IBSP* | integrin-binding sialoprotein | BE466675 | 4q22.1 | 5.6 |
| *CDC2* | cell division cycle 2, G1 to S and G2 to M | AL524035 | 10q21.2 | 5.5 |
| *IL8* | interleukin 8 | NM_000584 | 4q13.3 | 5.5 |
| *TEX15* | testis expressed sequence 15 | AL133653 | 8p12 | 5.5 |
|  | cDNA clone IMAGE:4454010 | BG165011 | 17q22 | 5.3 |
| *RHPN2* | rhophilin, Rho GTPase binding protein 2 | BG054987 | 19q13.11 | 5.3 |
| *CDCA2* | cell division cycle associated 2 | T90295 | 8p21.2 | 5.2 |
| *KIF14* | kinesin family member 14 | NM_014875 | 1q32.1 | 5.2 |
| *AKR1C1* | aldo-keto reductase family 1 member C1 | BC014579 | 10p15.1 | 5.1 |
| *ARL4C* | ADP-ribosylation factor-like 4C | BG435404 | 2q37.1 | 5.1 |
| *CCNB2* | cyclin B2 | NM_004701 | 15q22.2 | 5.0 |
| *BUB1* | BUB1 budding uninhibited by benzimidazoles 1 homolog (yeast) | AF043294 | 2q13 | 4.8 |
| *ANLN* | anillin, actin binding protein | AK023208 | 7p14.2 | 4.7 |
| *CDKN3* | cyclin-dependent kinase inhibitor 3 | AF213033 | 14q22.2 | 4.7 |
| *HIST1H1C* | histone 1, H1c | BC002649 | 6p22.1 | 4.7 |
| *KIF11* | kinesin family member 11 | NM_004523 | 10q23.33 | 4.7 |
| *PCDH19* | protocadherin 19 | AB037734 | Xq22.1 | 4.7 |
| *PVALB* | parvalbumin alpha | NM_002854 | 22q12.3 | 4.7 |
| *IQGAP3* | IQ motif containing GTPase activating protein 3 | AW271106 | 1q22 | 4.6 |
| *SHCBP1* | SHC SH2-domain binding protein 1 | NM_024745 | 16q11.2 | 4.6 |
| *AURKA* | aurora kinase A | NM_003158 | 20q13.2-q13.31 | 4.6 |
| *SYNGR3* | synaptogyrin 3 | NM_004209 | 16p13.3 | 4.6 |
| *HIST1H4H* | histone 1, H4h | NM_003543 | 6p22.1 | 4.5 |
| *RAB27B* | RAB27B, member RAS oncogene family | BF438386 | 18q21.2 | 4.5 |
| *TP73L* | tumor protein p73-like | AF091627 | 3q26.1 | 4.5 |
| *UBE2C* | ubiquitin-conjugating enzyme E2C | NM_007019 | 20q13.12 | 4.5 |
| *CASC5* | cancer susceptibility candidate 5 | BF248364 | 15q15.1 | 4.4 |
| *CENPF* | centromere protein F | U30872 | 1q41 | 4.4 |
| *HMMR* | hyaluronan-mediated motility receptor | NM_012485 | 5q34 | 4.4 |
| *NEK2* | NIMA (never in mitosis gene a)-related kinase 2 | NM_002497 | 1q32.3 | 4.4 |
| *PEG10* | paternally expressed 10 | BE858180 | 7q21.3 | 4.4 |
| *PHLDA1* | pleckstrin homology-like domain, family A, member 1 | AA576961 | 12q21.2 | 4.4 |
| *RGS7* | regulator of G-protein signalling 7 | NM_002924 | 1q43 | 4.4 |
| *SLC7A1* | solute carrier family 7 (cationic amino acid transporter, y+ system), member 1 | AA148507 | 13q12.3 | 4.4 |
|  | cDNA clone IMAGE:4824334 | BC024027 | Xp21.3 | 4.3 |
| *HIST1H2BD* | histone 1, H2bd | BC002842 | 6p22.1 | 4.3 |
| *MELK* | maternal embryonic leucine zipper kinase | NM_014791 | 9p13.2 | 4.2 |
| *PCDHB5* | protocadherin beta 5 | BC001186 | 5q31.3 | 4.2 |
| *TRIP13* | thyroid hormone receptor interactor 13 | NM_004237 | 5p15.33 | 4.2 |
| *TTK* | TTK protein kinase | NM_003318 | 6q14.1 | 4.2 |
| *EGLN3* | egl nine homolog 3 (C. elegans) | NM_022073 | 14q13.1 | 4.1 |
| *TRIM59* | tripartite motif-containing 59 | N90779 | 3q26.1 | 4.1 |
| *CENPA* | centromere protein A | NM_001809 | 2p23.3 | 4.0 |
| ^a^HUGO approved gene symbols are listed when known ^b^ Chromosomal location of the genes are listed ^c^FC is fold change; only genes with q<.05 and fold change>4 are listed | | | | |
