# Supplementary material for: Molecular signatures define two main classes of meningiomas
Source: Mol Cancer. 2007 Oct 15;6:64. doi: 10.1186/1476-4598-6-64 (PMC2173907; doi:10.1186/1476-4598-6-64)
Supplement: Additional file 2 — Genes reduced in grade 3 compared to grade 1 meningiomas. The data includes a list of genes with more than 4-fold reduction and q < 0.05 in expression levels in grade 3 compared to grade 1 meningiomas. [file 1476-4598-6-64-S2.doc]

| **Additional Table 2 - Genes reduced in grade 3 compared to grade 1 meningiomas** | | | | |
| --- | --- | --- | --- | --- |
| Gene Symbol^a^ | Gene Name | Accession Number | Chr Loc^b^ | FC^c^ |
| *SLC26A7* | solute carrier family 26, member 7 | Al758950 | 8q21.3 | 46.5 |
| *SFRP1* | secreted frizzled-related protein 1 | Al332407 | 8p11.21 | 20.5 |
|  | cDNA clone IMAGE:4795984 | BC036581 | 1p31.3 | 18.3 |
| *GPM6B* | glycoprotein M6B | AF016004 | Xp22.2 | 16.2 |
| *LPPR4* | plasticity related gene 1 | AW592563 | 1p21.2 | 16.2 |
| *TCEAL2* | transcription elongation factor A (SII)-like 2 | AF063606 | Xq22.1 | 15.2 |
|  | SAM domain containing 1 | Al700341 | 6q24.3 | 14.8 |
| *HSD17B2* | hydroxysteroid (17-beta) dehydrogenase 2 | NM_002153 | 16q23.3 | 13.4 |
| *PDZRN4* | PDZ domain containing RING finger 4 | NM_013377 | 12q12 | 13.4 |
|  | cDNA clone IMAGE:3086949 | BF509573 | 1q24.1 | 13.2 |
| *ANGPT1* | angiopoietin 1 | NM_001146 | 8q23.1 | 12.8 |
| *KCNMA1* | calcium-activated potassium channel subunit alpha 1 | Al129381 | 10q22.3 | 12.7 |
| *FBXO32* | F-box protein 32 | BF244402 | 8q24.13 | 11.1 |
| *NTRK2* | neurotrophic tyrosine kinase, receptor, type 2 | Al346341 | 9p21.33 | 10.9 |
| *LEPR* | leptin receptor | NM_002303 | 1p31.3 | 10.4 |
| *GPR88* | G-protein coupled receptor 88 | NM_022049 | 1p21.2 | 10.4 |
|  | hypothetical protein LOC340156 | BU675945 | 6p25.2 | 10.2 |
| *LPHN3* | latrophilin 3 | R50822 | 4q13.1 | 10.1 |
| *P2RY14* | purinergic receptor P2Y, G-protein coupled, 14 | NM_014879 | 3q25.1 | 9.9 |
|  | Transcribed locus, weakly similar to XP_520634.1 | BF724178 | 16q12.1 | 9.7 |
| *PENK* | Proenkephalin A precursor | NM_006211 | 8q12.1 | 9.5 |
|  | hypothetical protein LOC400120 | BG413606 | 13q13.3 | 9.3 |
| *AKR1B10* | aldo-keto reductase family 1, member B10 | NM_020299 | 7q33 | 9.1 |
| *LMO4* | LIM domain only 4 | R10289 | 1p22.3 | 9.0 |
| *ST8SIA1* | ST8 alpha-N-acetyl-neuraminide alpha-2,8-sialyltransferase 1 | L32867 | 12p12.1 | 8.9 |
| *C14orf140* | chromosome 14 open reading frame 140 | NM_024643 | 14q24.3 | 8.7 |
| *GPX3* | glutathione peroxidase 3 (plasma) | NM_002084 | 5q33.1 | 8.5 |
| *PDGFD* | platelet derived growth factor D | AB033832 | 11q22.3 | 8.3 |
| *PDE1C* | phosphodiesterase 1C | Al800515 | 7p15.1 | 8.2 |
| *IGFBP5* | insulin-like growth factor binding protein 5 | AW157548 | 2q35 | 8.1 |
|  | cDNA clone IMAGE:2620199 | AW149422 | 2q12.1 | 8.1 |
| *INMT* | indolethylamine N-methyltransferase | AF128846 | 7p15.1 | 7.9 |
| *SRPX* | sushi-repeat-containing protein, X-linked | NM_006307 | Xp11.4 | 7.9 |
| *GDF10* | growth differentiation factor 10 | NM_004962 | 10q11.22 | 7.8 |
| *COL12A1* | collagen, type XII, alpha 1 | AA788946 | 6q13 | 7.7 |
| *FBLN1* | fibulin 1 | NM_006486 | 22q13.31 | 7.7 |
| *HDAC9* | histone deacetylase 9 | BM726008 | 7p21.1 | 7.7 |
| *LMCD1* | LIM and cysteine-rich domains 1 | NM_014583 | 3p26.1 | 7.6 |
| *PAPPA* | pregnancy-associated plasma protein A | Al110886 | 9q33.1 | 7.6 |
| *HEPH* | hephaestin | NM_014799 | Xq12 | 7.4 |
| *RHOBTB3* | Rho-related BTB domain containing 3 | AK023621 | 5q15 | 7.4 |
|  | cDNA FLJ37310 fis, clone BRAMY2016706 | Al633559 | 6q26 | 7.1 |
| *TIMP3* | tissue inhibitor of metalloproteinases 3 | U67195 | 22q12.3 | 7.1 |
|  | hypothetical protein FLJ20701 | NM_017933 | 2q36.3 | 6.9 |
| *MCOLN3* | mucolipin 3 | Al636080 | 1p22.3 | 6.9 |
| *PLXNA2* | plexin A2 | NM_025179 | 1q32.2 | 6.8 |
| *RBP4* | retinol binding protein 4, plasma | NM_006744 | 10q23.33 | 6.8 |
|  | cDNA clone CS0DF032YA11 | AW953794 | 7p21.1 | 6.8 |
| *CYP1B1* | cytochrome P450, family 1, subfamily B, polypeptide 1 | AU154504 | 2p22.2 | 6.7 |
| *NR4A2* | nuclear receptor subfamily 4, group A, member 2 | S77154 | 2q24.1 | 6.7 |
| *RP13-347D8.3* | KIAA1210 protein | Al610999 | Xq24 | 6.6 |
| *PRRX1* | paired related homeobox 1 | AA775472 | 1q24.2 | 6.6 |
| *S100B* | S100 calcium binding protein B | BC001766 | 21q22.3 | 6.6 |
| *PDK4* | pyruvate dehydrogenase kinase, isoenzyme 4 | AV707102 | 7q21.3 | 6.5 |
| *C4B* | complement component 4B | NM_000592 | 6p21.32 | 6.4 |
| *ROR1* | receptor tyrosine kinase-like orphan receptor 1 | AK000776 | 1p31.3 | 6.3 |
| *KCNIP4* | Kv channel interacting protein 4 | Al732844 | 4p15.31 | 6.3 |
| *SERPINF1* | serpin peptidase inhibitor, clade F (alpha-2 antiplasmin, pigment epithelium derived factor), member 1 | NM_002615 | 17p13.3 | 6.2 |
|  | cDNA clone IMAGE:4293432 | BF671564 | 6q24.3 | 6.2 |
|  | cDNA DKFZp586G0321 | Al137566 | 11q22.1 | 6.2 |
| *RUNX2* | runt-related transcription factor 2 | AL353944 | 6p12.3 | 6.1 |
| *ITIH2* | inter-alpha (globulin) inhibitor H2 | NM_002216 | 10p14 | 6.1 |
|  | cDNA clone IMAGE:5276765 | BF059276 | 14q23.1 | 6.0 |
| *SYNPO2* | synaptopodin 2 | Al634580 | 4q26 | 6.0 |
|  | cDNA clone IMAGE:1698040 | Al143988 | 4q21.21 | 5.9 |
| *NNMT* | nicotinamide N-methyltransferase | NM_006169 | 11q23.2 | 5.9 |
| *BACH2* | BTB and CNC homology 2 | NM_021813 | 6q15 | 5.8 |
|  | cDNA clone IMAGE:4605252 | BG435302 | 5q33.3 | 5.8 |
| *BCAR3* | breast cancer anti-estrogen resistance 3 | NM_003567 | 1p22.1 | 5.7 |
| *GPR27* | G protein-coupled receptor 27 | Al703476 | 3p13 | 5.7 |
| *NLGN1* | neuroligin 1 | NM_014932 | 3q26.31 | 5.6 |
| *SCN2B* | sodium channel, voltage-gated, type II, beta | AA447729 | 11q23.3 | 5.6 |
| *CNTN4* | contactin 4 | R42166 | 3p26.3 | 5.5 |
| *MMP28* | matrix metallopeptidase 28 | NM_024302 | 17q12 | 5.5 |
| *MN1* | meningioma (disrupted in balanced translocation) 1 | NM_002430 | 22q12.1 | 5.5 |
| *PLAC9* | placenta-specific 9 | AW964972 | 10q22.3 | 5.4 |
| *CPXM2* | carboxypeptidase X (M14 family), member 2 | BF726934 | 10q26.13 | 5.4 |
| CYR61 | cysteine-rich, angiogenic inducer, 61 | AF003114 | 1p22.3 | 5.4 |
| *RASGRF2* | Ras protein-specific guanine nucleotide-releasing factor 2 | Al912976 | 5q14.1 | 5.4 |
| *CPE* | carboxypeptidase E | Al922855 | 4q32.3 | 5.3 |
| *DDX43* | DEAD (Asp-Glu-Ala-Asp) box polypeptide 43 | NM_018665 | 6q13 | 5.3 |
| *HPGD* | 15-hydroxyprostaglandin dehydrogenase | J05594 | 4q34.1 | 5.3 |
| *VCAM1* | vascular cell adhesion molecule 1 | NM_001078 | 1p21.2 | 5.3 |
| *CD200* | CD200 molecule | AF063591 | 3q13.2 | 5.2 |
|  | hypothetical protein LOC143381 | AW263087 | 10q25.3 | 5.2 |
|  | hypothetical protein MGC45438 | Al823572 | 16p13.3 | 5.1 |
| *AEBP1* | AE binding protein 1 | NM_001129 | 7p13 | 5.0 |
| *CACNA1D* | calcium channel, voltage-dependent, L type, alpha 1D subunit | BE550599 | 3p21.1 | 5.0 |
|  | cDNA clone IMAGE:3439264 | BF939996 | 8q11.23 | 5.0 |
| *GPR177* | G protein coupled receptor 177 | AL534095 | 1p31.3 | 5.0 |
| *FILIP1* | filamin A interacting protein 1 | AL832009 | 6q14.1 | 5.0 |
| *SLC1A4* | solute carrier family 1 (glutamate/neutral amino acid transporter), member 4 | BF340083 | 2p14 | 5.0 |
| *MAL2* | mal, T-cell differentiation protein 2 | AL117612 | 8q24.12 | 4.9 |
| *AFF2* | AF4/FMR2 family, member 2 | NM_002025 | Xq28 | 4.9 |
| *MT1M* | metallothionein 1M | R06655 | 16q13 | 4.9 |
| *ARID5B* | AT-rich interactive domain 5B (MRF1-like) | BG285011 | 10q21.2 | 4.8 |
| *DLL1* | delta-like 1 (Drosophila) | AF196571 | 6q27 | 4.8 |
| *STON2* | stonin 2 | BE379761 | 14q31.1 | 4.8 |
| *CALML4* | calmodulin-like 4 | AW025529 | 15q23 | 4.7 |
| *EFS* | embryonal Fyn-associated substrate | NM_005864 | 14q11.2 | 4.7 |
| *FOSB* | FBJ murine osteosarcoma viral oncogene homolog B | NM_006732 | 19q13.32 | 4.7 |
| *NEDD9* | Neural precursor cell expressed, developmentally down-regulated 9 | AK000850 | 6p24.1 | 4.7 |
| *PRKAR2B* | protein kinase, cAMP-dependent, regulatory, type II, beta | NM_002736 | 7q22.3 | 4.7 |
|  | CDNA clone IMAGE:5312689 | Al479277 | 6q14.1 | 4.6 |
| *C14orf39* | chromosome 14 open reading frame 39 | AL832219 | 14q23.1 | 4.6 |
| *EGFL6* | EGF-like-domain, multiple 6 | NM_015507 | Xp22.2 | 4.6 |
| *IL1R1* | interleukin 1 receptor, type I | NM_000877 | 2q12.1 | 4.6 |
| *SLC16A9* | solute carrier family 16 (monocarboxylic acid transporter 9) | BG401568 | 10q21.1 | 4.6 |
| *C1QTNF7* | C1q and tumor necrosis factor related protein 7 | BE856929 | 4p15.33 | 4.5 |
| *CPM* | carboxypeptidase M | NM_001874 | 12q15 | 4.5 |
| *COL8A1* | collagen, type VIII, alpha 1 | BE877796 | 3q12.1 | 4.5 |
| *MBP* | myelin basic protein | AW070431 | 18q23 | 4.5 |
| *SSPN* | sarcospan (Kras oncogene-associated gene) | NM_005086 | 12p12.1 | 4.5 |
| *C1QDC2* | C1q domain containing 2 | BF589994 | 1p36.33 | 4.4 |
| *HPR* | haptoglobin-related protein | NM_020995 | 16q22.3 | 4.4 |
| *LRRN1* | leucine rich repeat neuronal 1 | N71874 | 3p26.2 | 4.4 |
| *PLP1* | proteolipid protein 1 (Pelizaeus-Merzbacher disease, spastic paraplegia 2, uncomplicated) | BC002665 | Xq22.2 | 4.3 |
| *SMOC2* | SPARC related modular calcium binding 2 | AB014737 | 6q27 | 4.3 |
| *SNED1* | sushi, nidogen and EGF-like domains 1 | N73970 | 2q37.3 | 4.2 |
| *SVEP1* | sushi, von Willebrand factor type A, EGF and pentraxin domain containing 1 | AA716107 | 9q31.3 | 4.2 |
| *C6orf117* | chromosome 6 open reading frame 117 | AA418816 | 6q14.3 | 4.1 |
| *GRIA3* | glutamate receptor, ionotrophic, AMPA 3 | BC032004 | Xq25 | 4.1 |
| *PGR* | progesterone receptor | NM_000926 | 11q22.1 | 4.1 |
| *JUN* | v-jun avian sarcoma virus 17 oncogene homolog | BC002646 | 1p32.1 | 4.0 |
| *LTBP2* | latent transforming growth factor beta binding protein 2 | NM_000428 | 14q24.3 | 4.0 |
| *RBM11* | RNA binding motif protein 11 | N94835 | 21q11.2 | 4.0 |
| ^a^HUGO approved gene symbols are listed when known ^b^ Chromosomal location of the genes are listed  ^c^FC is fold change; only genes with q<.05 and fold change>4 are listed | | | | |
